# Is MASLD Not Just a Liver Disease? Bidirectional Gut–Liver Crosstalk as a Driver of Chronic Liver Disease

**DOI:** 10.3390/life16071076

**Published:** 2026-06-27

**Authors:** Iulia Cristina Marginean, Sergiu Marian Cazacu, Cristina Maria Marginean, Mihaela Popescu, George Alexandru Iacob, Marian Sorin Popescu, Cristin Constantin Vere

**Affiliations:** 1 Doctoral School, University of Medicine and Pharmacy of Craiova, 200349 Craiova, Romania; iulia.cristina18@yahoo.com; 2 Research Center of Gastroenterology and Hepatology, University of Medicine and Pharmacy of Craiova, 200349 Craiova, Romania; vere_cristin@yahoo.com; 3 Department of Internal Medicine, University of Medicine and Pharmacy of Craiova, 200349 Craiova, Romania; popescu.mariansorin@yahoo.com; 4 Department of Endocrinology, University of Medicine and Pharmacy of Craiova, 200349 Craiova, Romania; mihaela.n.popescu99@gmail.com; 5 Department of Radiology and Medical Imaging, Clinical Emergency County Hospital Craiova, 200349 Craiova, Romania; georgeicb5@gmail.com

**Keywords:** MASLD, IBS, IBS-D, gut-liver axis, intestinal permeability, bile acids, TLR4

## Abstract

Irritable bowel syndrome (IBS) and metabolic dysfunction-associated steatotic liver disease (MASLD) are two of the most common gastroenterological conditions worldwide. Traditionally viewed as unrelated, with one serving a canonical functional role and the other a purely metabolic function, these two processes have recently been linked by compelling evidence, challenging their traditional segregation and pointing to a significant, biologically relevant association. This review aims to evaluate the current evidence for a potential causal contribution of IBS to hepatic steatosis, critically examining the proposed pathophysiological mechanisms via the gut–liver axis while acknowledging that the available data are primarily observational. Notably, epidemiological studies demonstrate a 1.4–2.0-fold increased association between IBS and MASLD, independent of obesity and metabolic syndrome, though causality remains to be established. The primary mechanism is increased intestinal permeability (“leaky gut”) leading to endotoxemia, activation of hepatic toll-like receptor 4 (TLR4) receptors, and subsequent de novo lipogenesis. The relationship is bidirectional, with steatosis also worsening gut barrier function. Therefore, we highlight emerging evidence suggesting that irritable bowel syndrome, particularly the diarrhea-predominant subtype (IBS-D), may contribute to hepatic steatosis through plausible biological mechanisms, though direct causal evidence in humans remains limited. Accordingly, routine screening for metabolic dysfunction-associated steatotic liver disease (MASLD) may be warranted in patients with long-standing IBS-D.

## 1. Introduction

Epidemiological evidence indicates a significant, positive association between Irritable Bowel Syndrome (IBS) (10–15% global prevalence) and Metabolic Dysfunction-Associated Steatotic Liver Disease (MASLD) (25–30% prevalence). Patients with one condition are frequently found to have higher rates of the other, suggesting either a bidirectional relationship, shared risk factors, or common underlying pathophysiological pathways [1,2]. However, the observational nature of most studies precludes definitive causal inference. This link is driven by shared mechanisms of metabolic syndrome, both conditions sharing risk factors like obesity, diabetes, hypertension, dyslipidemia, and gut microbiota dysbiosis [3]. This bidirectional network integrates signals derived from the intestinal microbiota, dietary factors, and host immune responses, which collectively influence hepatic metabolism, inflammation, and fibrogenesis. Disruptions in gut microbiota composition (dysbiosis), increased intestinal permeability, and translocation of microbial products such as lipopolysaccharides (LPS) contribute to chronic low-grade inflammation and liver injury. Understanding the mechanistic interplay within the gut–liver axis is therefore critical to redefining MASLD beyond a purely hepatic disorder and to identifying novel therapeutic targets.

## 2. Materials and Methods

A literature search was conducted in PubMed, Scopus, and Web of Science from January 2000 to April 2026. Search terms included terms found in title/abstract (tiab) and Medical Subject Headings (MeSH): “MASLD” [tiab], “NAFLD” [tiab], “MASH” [tiab], “NASH” [tiab], “gut–liver axis” [tiab], “gut–liver crosstalk” [tiab], “gut microbiota” [tiab], “dysbiosis” [tiab], “intestinal permeability” [tiab], “bacterial translocation” [tiab], “endotoxemia” [tiab], “LPS” [tiab], “bile acids” [tiab], “FXR” [tiab], “short-chain fatty acids” [tiab], “liver fibrosis” [tiab], “cirrhosis” [tiab], “chronic liver disease” [tiab], “irritable bowel syndrome” [tiab], “IBS” [tiab], “functional gastrointestinal disorder” [tiab], “Non-alcoholic Fatty Liver Disease” [MeSH], “Gastrointestinal Microbiome” [MeSH], “Dysbiosis” [MeSH], “Bacterial Translocation” [MeSH], “Lipopolysaccharides” [Mesh], “Intestinal Mucosa” [Mesh], “Liver Cirrhosis” [Mesh], “Inflammation” [Mesh], “Bile Acids and Salts” [MeSH], “Fatty Acids, Volatile” [MeSH], “Irritable Bowel Syndrome” [MeSH]. Studies were considered for inclusion if they met the following criteria: original research articles (including cross-sectional, cohort, case–control, and randomized controlled trial designs); systematic reviews and meta-analyses; narrative reviews providing comprehensive overviews of the topic; clinical guidelines and consensus statements; mechanistic studies (in vitro, ex vivo, or animal models) directly relevant to gut–liver axis pathways in the context of IBS or MASLD; studies published between January 2000 and April 2026. The exclusion criteria were studies classified as case reports, case series, conference abstracts without accompanying full-text publications, editorials, commentaries, and opinion pieces, studies focused exclusively on pediatric populations (unless directly relevant to mechanistic pathways) or studies of other liver diseases (e.g., viral hepatitis, autoimmune hepatitis, alcoholic liver disease) without specific relevance to MASLD or IBS. This preliminary search was deliberately broad to encompass the full scope of the topic. Subsequently, the reference lists of the retrieved articles were manually screened to identify additional relevant publications not captured by the primary search, thereby ensuring the comprehensive and organic incorporation of both foundational and recent studies. As this is a narrative review, study selection was based on relevance to gut–liver crosstalk mechanisms and clinical management, and no formal risk-of-bias assessment was performed. All records identified through database searches were exported to Zotero (version 6.0.37), and duplicates were removed electronically. Titles and abstracts were screened independently for relevance to the gut–liver axis, IBS, and/or MASLD. Full-text articles were retrieved for potentially relevant records and assessed against the predefined inclusion/exclusion criteria. Studies deemed eligible were included in the review. Reference lists of included articles and relevant reviews were manually screened to identify additional studies not captured by the primary search. The manuscript was drafted and prepared using Microsoft Word (version 16.59). All graphical figures were created using Affinity Designer (version 2.6.4) and Draw.io (version 29.2.9). Reference management, including the storage, organization, and citation of all the literature, was handled using Zotero (version 6.0.37). The final manuscript underwent proofreading and language refinement assisted by the AI language model DeepSeek (version V3).

## 3. Gut–Liver Axis as a Unifying Concept

Altered gut flora can contribute to both IBS symptoms and liver fat accumulation. Increased gut permeability (“Leaky Gut”) is seen in both IBS and MASLD, allowing toxins to travel via the portal vein to the liver, enhancing inflammation. Alterations in bile acid signaling, often found in irritable bowel syndrome with diarrhea (IBS-D) patients, correlate with increased hepatic fat and severity of fibrosis in MASLD patients [4,5].

Purssell’s study in 2023 found that 35.2% of patients with MASLD met the criteria for Rome IV IBS, a rate nearly ten times higher than in the general population [6]. A 2025 study conducted by Zhou Y et al. highlighted that metabolic dysfunction-associated steatotic liver disease (MASLD), including “pure MASLD” and “MASLD with moderate alcohol use” (MetALD), is associated with a 11–30% elevated risk of IBS, increasing with the number of cardiometabolic risk factors [2].

The gut–liver axis has been proposed as a unifying conceptual framework, raising the hypothesis that IBS and early MASLD may represent phenotypic variants of a shared pathological continuum. However, this remains a hypothesis requiring prospective validation, as current evidence cannot definitively establish whether the observed association reflects shared pathophysiology, causal directionality, or confounding by common risk factors. The discriminating factor is individual susceptibility of the intestine (leading to IBS) versus the hepatocyte (leading to steatosis), driven by a common upstream dysbiosis and barrier defect [2].

But IBS was viewed in the old paradigm as a “functional” disorder of pain and motility, with inflammation ignored or minimized, while MASLD was viewed as a “metabolic” storage disease from excess calories, with the gut merely a secondary factor; therefore, IBS patients’ steatosis is missed, and MASLD patients’ IBS symptoms are dismissed as “functional overlay” [3].

Gut dysbiosis, marked by an increase in *Ruminococcus gnavus* and a decrease in *Faecalibacterium prausnitzii*, drives two parallel processes: fermentation, causing gas, bloating, and abdominal pain, and altered choline metabolism, reducing phosphatidylcholine synthesis. This choline-derived defect may impair very low-density lipoprotein (VLDL) export from the liver, representing a plausible mechanism that could contribute to the MASLD phenotype, though direct evidence in IBS patients remains limited [3,4].

Increased intestinal permeability (“leaky gut”) promotes submucosal mast cell activation, leading to visceral hypersensitivity. Concurrently, bacterial products (e.g., LPS) drive portal endotoxemia, activating toll-like receptor 4 (TLR4) on hepatic stellate cells and hepatocytes. Altered bile acid metabolism further contributes: in the colon, bile acids activate Takeda G protein-coupled receptor 5 (TGR5) and farnesoid X receptor (FXR), accelerating transit and causing diarrhea; in the liver, bile acids inhibit FXR, thereby promoting de novo lipogenesis (DNL) [7,8]. Low-grade inflammation releases interleukin-6 (IL-6) and tumor necrosis factor-alpha (TNF-α), driving both neuronal sensitization (pain) and insulin resistance with hepatic lipid accumulation. Depending on host genetics, diet, and mucosal injury severity, the same LPS can either cause pain and diarrhea via colonic afferents or promote fatty liver via portal circulation [7].

### Does IBS Contribute to Steatosis via the Gut–Liver Axis? A Mechanistic Hypothesis

**Figure 1:**
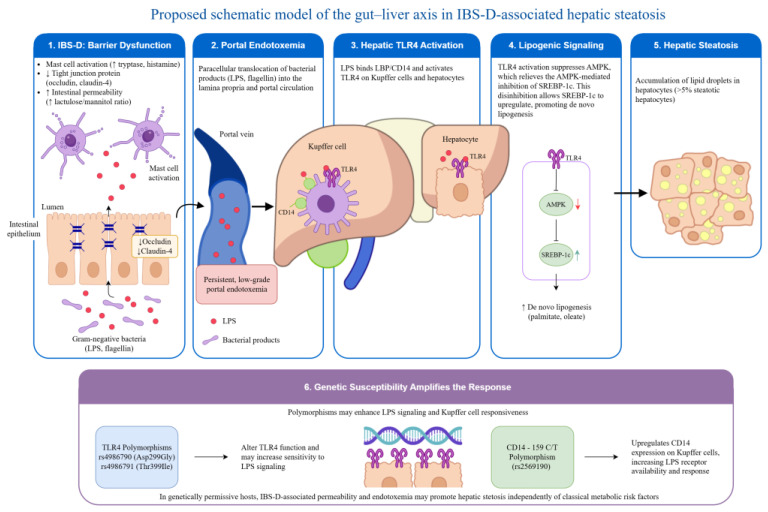
Proposed schematic model of the gut–liver axis in IBS-D-associated hepatic steatosis.AMPK, AMP-activated protein kinase; CD14, cluster of differentiation 14; IBS-D, irritable bowel syndrome with diarrhea; LBP, lipopolysaccharide-binding protein; LPS, lipopolysaccharide; SREBP-1c, sterol regulatory element-binding protein 1c; TLR4, toll-like receptor 4.

A plausible mechanistic hypothesis, based on human clinical evidence proposes that IBS (specifically IBS-D, increased permeability subtype) may generate persistent, low-grade portal endotoxemia sufficient to induce hepatic steatosis in a genetically permissive host, potentially independent of classical metabolic risk factors. In IBS-D (diagnosed by Rome IV criteria) there is robust evidence of increased mast cell activation (tryptase, histamine) in terminal ileum/colon, that decrease occludin and claudin-4 expression, leading to an increased intestinal permeability (confirmed by lactulose/mannitol ratio, along with paracellular translocation of Gram-negative bacterial products (LPS, flagellin) [7,8]. This translocation may drive portal vein endotoxemia, which could activate hepatocyte TLR4, suppress AMP-activated protein kinase (AMPK), and upregulate sterol regulatory element-binding protein 1c (SREBP-1c) to enhance DNL (palmitate, oleate), ultimately producing hepatic steatosis (>5% of hepatocytes containing lipid droplets) [8]. This mechanistic model, while biologically plausible and supported by in vitro and animal data, has not been definitively proven in human subjects. Most supportive evidence derives from observational studies, experimental models, and extrapolation from other disease states. Direct demonstration that IBS-induced endotoxemia causes hepatic steatosis in humans would require prospective interventional studies that have not yet been performed. Preliminary genetic association studies suggest that IBS patients who develop steatosis may show enrichment for TLR4 polymorphisms (rs4986790, rs4986791) that confer LPS hyper-responsiveness [7,8], along with the CD14-159 C/T polymorphism, which upregulates LPS receptor expression on Kupffer cells [9]. However, these findings are based on small sample sizes and require replication in larger, well-powered cohorts. The functional significance of these polymorphisms in the specific context of IBS-MASLD comorbidity remains speculative. These specific Single Nucleotide Polymorphisms (SNPs) are associated with modified TLR4 function, which acts as a receptor for LPS from Gram-negative bacteria. CD14-159 C/T polymorphism, located in the promoter of the CD14 gene (a co-receptor for TLR4), is linked to increased CD14 receptor expression on Kupffer cells [9]. Notably, these genetic variants have been studied primarily in other disease contexts (e.g., sepsis, inflammatory bowel disease), and their role in IBS-MASLD specifically remains an area of active investigation rather than established fact. The combination of increased LPS presence (often due to altered permeability/dysbiosis in IBS) and increased sensitivity to LPS (due to TLR4/CD14 variants) activates Kupffer cells, that produce pro-inflammatory cytokines and therefore contribute to fat accumulation and inflammation in the liver. It is hypothesized that IBS patients without these variants do not usually develop steatosis despite identical IBS severity and permeability. Prospective studies with larger sample sizes and functional validation are needed to determine the clinical significance of these polymorphisms in IBS-associated liver injury [8,9]. To illustrate this proposed sequence of events, we present a schematic model of the gut–liver axis in IBS-D-associated hepatic steatosis (Figure 1).

## 4. Bidirectional Association Between IBS and MASLD

### 4.1. Cross-Sectional Evidence: Increased MASLD Prevalence Among IBS Patients

Based on systematic reviews and several large-scale studies, there is consistent evidence that patients with IBS have a significantly higher prevalence of MASLD compared to those without IBS. Thus, the association is not unidirectional; rather, patients with one condition are at increased risk for the other, suggesting shared pathophysiological mechanisms.

Table 1 summarizes evidence of MASLD prevalence in IBS patients.

Bidirectionality characterizes the relationship—MASLD frequently coexists with IBS, and conversely, MASLD itself independently predicts future development of IBS symptoms [4]. Shared risk factors cannot fully explain the IBS–MASLD relationship. While obesity and metabolic syndrome are common to both conditions, the studies cited above adjusted for these factors and still observed a significant independent association, indicating a direct connection beyond metabolic confounders [10]. Therefore, it is hypothesized that dysbiosis, enhanced intestinal permeability, and sustained low-grade inflammation constitute the core pathophysiological pathways underlying the bidirectional association between IBS and MASLD.

### 4.2. Temporal Directionality: Evidence That IBS May Precede Steatosis—Early but Promising Findings

Emerging evidence indicates [12] that IBS symptoms and underlying gut dysfunction may precede or correlate with the development of hepatic steatosis [4,13,14]. IBS-D shows the strongest association with hepatic steatosis, driven by bidirectional communication between the gut microbiota and the liver. In IBS-D, multiple disruptions to the gut–liver axis converge to create a “perfect storm” that stresses the liver and promotes fat accumulation. These disruptions are generally more severe than in irritable bowel syndrome with constipation (IBS-C) or mixed irritable bowel syndrome (IBS-M). Furthermore, small intestinal bacterial overgrowth (SIBO) is more common in IBS-D, providing a potential source of the increased bacterial products (e.g., LPS) that may drive hepatic inflammation [15,16,17,18]. Whether this SIBO-associated endotoxemia translates clinically to hepatic steatosis in IBS-D patients requires prospective investigation.

### 4.3. Metabolic Syndrome Components as Potential Confounders in IBS Studies

There is a growing body of research directly investigating the relationship between metabolic syndrome components and IBS (Table 2), highlighting that IBS is associated with a significantly increased prevalence of metabolic syndrome. Furthermore, emerging data suggest the existence of overlapping pathophysiological mechanisms [19,20,21]. However, the presence of this association raises an important question: does IBS independently contribute to MASLD, or is the observed relationship primarily driven by shared metabolic and lifestyle determinants?

#### 4.3.1. Shared Risk Factors and the Problem of Residual Confounding

Several factors that predispose to MASLD are also overrepresented in IBS populations, potentially creating a spurious association:

Dietary Patterns: The Western diet—characterized by high intake of ultra-processed foods, refined carbohydrates, and saturated fats—is a well-established risk factor for both obesity and MASLD [24,25]. In IBS patients, dietary habits may be further modified by symptom-related food avoidance, which can paradoxically lead to either increased consumption of low-fermentable oligosaccharides, disaccharides, monosaccharides, and polyols (FODMAP) but high-fat foods or nutritional inadequacies that affect gut barrier function [26]. The low-FODMAP diet, while effective for IBS symptom control, may reduce prebiotic fiber intake and alter the gut microbiome in ways that could influence metabolic health [27]. However, few studies have rigorously controlled for dietary intake when examining the IBS–MASLD association.

Physical Inactivity: Sedentary behavior is more common among individuals with chronic gastrointestinal symptoms, including IBS, due to symptom-related activity avoidance, fatigue, or psychological distress [28]. Physical inactivity independently promotes hepatic steatosis through reduced fatty acid oxidation and increased insulin resistance [29]. Few IBS–MASLD studies have objectively measured or adequately adjusted for physical activity levels.

Psychiatric Comorbidities: Anxiety and depression are highly prevalent in IBS (affecting up to 40–60% of patients) [30] and are independently associated with MASLD through mechanisms including hypothalamic–pituitary–adrenal (HPA) axis dysregulation, altered eating behaviors, and medication effects [31,32]. Psychotropic medications (e.g., antidepressants, antipsychotics) can themselves contribute to weight gain and metabolic dysfunction [33]. The potential for psychiatric comorbidity and its treatment to confound the IBS–MASLD relationship has not been systematically examined.

Sleep Disturbances: Poor sleep quality is common in IBS and independently associated with metabolic dysfunction, insulin resistance, and hepatic steatosis [34]. The role of sleep as a confounder or mediator in the IBS–MASLD association remains unexplored.

Medication Use: IBS patients frequently use medications that may affect metabolic parameters, including: antidepressants (selective serotonin reuptake inhibitors, serotonin and norepinephrine reuptake inhibitors, tricyclic antidepressants), that lead to weight gain, metabolic changes [35]; proton pump inhibitors, that modify gut microbiome, with potential metabolic effects [36]; laxatives, antidiarrheals, with possible effects on nutrient absorption and microbiome composition.

#### 4.3.2. Evidence for Independence Beyond Confounders

Despite the substantial potential for confounding, several lines of evidence suggest that the IBS–MASLD association may not be entirely explained by shared risk factors, such as the adjustment for metabolic factors. The large-scale study by Sarmini et al. (2019) [10] adjusted for age, obesity, and diabetes and still observed a 3.2-fold increased odds of MASLD in IBS patients (95% CI: 3.13–3.28). Similarly, Wu et al. (2022) [1] adjusted for body mass index, metabolic syndrome components, and lifestyle factors, finding a persistent 13% increased risk of IBS in patients with pre-existing MASLD. While these adjustments strengthen the case for an independent association, residual confounding cannot be entirely excluded, as dietary intake and physical activity were not rigorously measured in these large administrative datasets. Another line of evidence suggest a Subtype Specificity association, that appears strongest for IBS-D, which is less consistently associated with obesity than other IBS subtypes [18]. If obesity were the primary driver, one might expect a stronger association with IBS-C or IBS-M, given their higher prevalence of constipation and potential association with metabolic syndrome [37]. The subtype specificity of the association supports a mechanism beyond simple metabolic confounding. Furthermore, temporal evidence is highlighted by the prospective cohort study by Wu et al. (2022) [1], proving that MASLD predicted future IBS development over ~12 years, with risk increasing with fatty liver severity. This temporal relationship, while not proving causality, is less consistent with a purely confounding explanation, as confounders would be expected to be present at both time points. However, the study did not have repeated measures of diet, physical activity, or psychological factors, limiting its ability to fully account for time-varying confounders.

The bidirectional nature of the relationship makes it difficult to determine whether IBS precedes MASLD or whether early, subclinical metabolic dysfunction contributes to IBS symptoms.

## 5. Mechanistic Pathways in IBS

### 5.1. Anatomical and Physiological Basis of Gut–Liver Axis

In the context of IBS, the gut–liver axis is of critical pathophysiological relevance. The liver receives approximately 70–75% of its blood supply from the gastrointestinal tract via the portal vein, rendering it uniquely susceptible to alterations in intestinal barrier function and the luminal microenvironment. Specifically, increases in intestinal permeability, shifts in gut microbial composition (dysbiosis), and changes in the profile of microbe-derived metabolites—such as short-chain fatty acids, bile acids, and LPS—can directly influence hepatic immunological and metabolic responses [38,39]. These gut-derived signals may, in turn, perpetuate low-grade inflammation, modulate visceral sensitivity, and contribute to the multifaceted symptomatology of IBS, highlighting the gut–liver axis as a potential therapeutic target [38,39].

A key mechanism linking the gut and liver in IBS involves the interaction between gut bacteria and bile acids [40]. Bile acids, synthesized in the liver to facilitate lipid digestion, undergo significant chemical modification by the gut microbiota upon reaching the intestinal lumen [40]. This microbial–bile acid crosstalk plays a critical role in shaping the composition and metabolic activity of the intestinal microbial ecosystem. In IBS, this finely tuned interaction becomes disrupted, leading to an altered bile acid profile and a perturbed gut microbial environment [41]. Dysfunctional bile acid signaling can contribute to the hallmark symptoms of IBS, such as diarrhea or constipation, because bile acids influence intestinal motility and fluid secretion [41].

Modern research has expanded the concept into a “tri-directional” system involving the gut, liver, and brain, often called the gut–liver–brain axis (GLBA). This is particularly relevant for IBS, which is classified as a disorder of gut–brain interaction [42]. Psychological stress activates the hypothalamic–pituitary–adrenal (HPA) axis, releasing cortisol. When the gut barrier is compromised, bacterial products like LPS can travel via the portal vein to the liver. This triggers low-grade inflammation in the liver and sends signals back to the brain, perpetuating a cycle of stress and gastrointestinal symptoms [42].

### 5.2. Increased Intestinal Permeability (“Leaky Gut”) in IBS

A considerable subset of patients—notably those with post-infectious IBS (PI-IBS) and IBS-D—demonstrate objectively quantifiable increases in intestinal permeability, commonly referred to as “leaky gut”.

The intestinal barrier is a multilayer system: the mucus layer, epithelial cells, tight junction (TJ) complexes, and the underlying immune compartment (lamina propria) [43,44]. For the purposes of this review, increased intestinal permeability is defined specifically as enhanced paracellular flux resulting from dysfunctional TJs, as opposed to transcellular transport.

The intestinal epithelium is a single cell layer connected by TJ proteins (e.g., claudins, occludin, zonula occludens-1 (ZO-1)). These TJs regulate paracellular transport, allowing small molecules to pass while excluding larger antigens, bacteria, and bacterial products. Increased intestinal permeability led to pathological loosening of TJs, resulting in enhanced paracellular flux of luminal contents into the lamina propria and systemic circulation.

Multiple converging pathways contribute to the disruption of TJ integrity. Among these, mast cell activation is particularly prominent in IBS-D. In affected individuals, mucosal mast cell counts are elevated. The mast cell-derived protease tryptase activates protease-activated receptor-2 (PAR-2) on enterocytes, thereby triggering myosin light chain kinase (MLCK). This, in turn, induces contraction of the perijunctional actin cytoskeleton and promotes TJ opening. Concurrently, mast cell-derived histamine reduces transepithelial electrical resistance (TER) and stimulates visceral afferent nerves [43,44].

Low-grade inflammation, which is predominantly observed in PI-IBS, is characterized by the persistent elevation of pro-inflammatory cytokines—including TNF-α, interferon-gamma (IFN-γ), and interleukin-1 beta (IL-1β)—following an acute enteric infection. Mechanistically, TNF-α downregulates the expression of occludin, a key structural protein of TJs, while IFN-γ impairs TJ assembly through activation of the nuclear factor kappa-B (NF-κB) signaling pathway [43,44,45].

IBS-associated dysbiosis (reduced *Bifidobacterium*, increased *Ruminococcus gnavus*) reduces butyrate production, a key TJ-strengthening short-chain fatty acid [45]. Increased bile acids (common in IBS-D) activate mast cells directly. Chronic stress elevates corticotropin-releasing hormone (CRH), which acts on enteric neurons and mast cells to increase permeability [46].

Supporting evidence includes an elevated lactulose/mannitol (L/M) excretion ratio indicating increased paracellular permeability, raised circulating and fecal zonulin levels as a marker of tight junction dysregulation, and upregulation of the pore-forming protein claudin-2, which promotes enhanced sodium and water flux across the intestinal epithelium.

Zonulin, a haptoglobin precursor binds to C-X-C motif chemokine receptor 3 (CXCR3) on enterocytes, triggering a signaling cascade (via phospholipase C and protein kinase C alpha) that leads to reversible opening of the paracellular pathway. Because of its specific role, zonulin has been extensively investigated as a potential biomarker of increased intestinal permeability in IBS [47]. It is the only known physiological modulator of intercellular tight junctions, and its upregulation has been implicated in impaired intestinal barrier function. Sturgon and Fasano, in a comprehensive review, outlined how zonulin-mediated increases in intestinal permeability facilitate the uncontrolled translocation of dietary and microbial antigens across the mucosal barrier, thereby promoting a shift from immune tolerance toward a pro-inflammatory state. This mechanism is thought to underpin the pathogenesis of various chronic inflammatory conditions, providing a plausible link between gut barrier integrity and systemic inflammation [48].

Multiple studies have documented alterations in zonulin levels among patients with irritable bowel syndrome (IBS). Specifically, serum zonulin concentrations are significantly elevated in individuals with IBS compared to healthy controls, reaching levels comparable to those observed in active celiac disease [49]. Furthermore, fecal zonulin correlates positively with abdominal pain severity, diarrhea frequency, and overall disease severity in IBS patients [50]. Among IBS subtypes, patients with IBS-D and PI-IBS show the most consistent elevations across both serum and fecal measurements [50]. In a cohort of patients with IBS, Gaus and Livzan demonstrated that fecal zonulin levels are closely associated with dietary habits, anxiety levels, and the secretion of cortisol and serotonin, and are also correlated with the severity of abdominal pain and diarrhea [51].

The L/M ratio is a well-established, non-invasive dual-sugar test for assessing intestinal permeability, but its role in IBS remains primarily within research settings [52]. This assay relies on the differential absorption of two orally administered sugar probes. Mannitol, a small monosaccharide, is passively absorbed through small aqueous channels located at the villous tip, primarily via the transcellular route. In contrast, lactulose, a larger disaccharide, traverses the intestinal epithelium via the paracellular pathway through larger gaps present at the villous base and within the crypts. An elevated urinary L/M ratio therefore reflects increased intestinal permeability, specifically enhanced paracellular flux [39]. Findings regarding intestinal permeability in patients with IBS have been heterogeneous across studies. Nevertheless, the most compelling evidence supports the presence of increased permeability in specific clinical subgroups, particularly those with IBS-D. In this population, multiple studies have demonstrated significantly elevated mucosal permeability in both the small intestine and the colon compared to healthy control subjects [53].

Among patients with IBS-D, the identification of those exhibiting an altered L/M ratio may define a distinct pathophysiological subtype. These individuals typically present with elevated levels of inflammatory markers, increased intestinal fatty acid-binding protein (I-FABP)—a surrogate marker of enterocyte damage—and evidence of bacterial translocation, thereby supporting the notion of a more pronounced disruption of gut barrier integrity in this subgroup.

Furthermore, familial influences on gut permeability have been documented, as siblings and parents of children with IBS exhibit an increased baseline small intestinal L/M ratio, suggesting a potential heritable component to intestinal barrier dysfunction [54]. Despite these associations, the clinical utility of the L/M ratio is constrained by several significant confounding factors, including marked day-to-day intraindividual variability, geographical differences in baseline permeability values, and dependence on patient hydration status and total urine volume [54]. Consequently, while the L/M ratio remains a valuable research tool for elucidating disease mechanisms and phenotyping patients in mechanistic studies, its routine application in clinical practice cannot be recommended until standardized testing protocols and clearly defined therapeutic correlates have been established [55,56].

Multiple studies have consistently demonstrated that patients with IBS-D exhibit significantly increased expression of claudin-2 in both the colon and small intestine compared to healthy controls [57,58]. This upregulation of the pore-forming tight junction protein claudin-2 is mechanistically attributed to post-transcriptional dysregulation involving microRNAs. Specifically, the downregulation of hsa-miR-16—a microRNA that normally represses claudin-2 translation under physiological conditions—leads to subsequent overexpression of claudin-2 at the protein level [59,60]. Furthermore, this molecular alteration is associated with the activation of mucosal mast cells, and its expression level correlates with the severity of clinical symptoms and barrier dysfunction in IBS-D patients, reinforcing its role in disease pathogenesis [57].

The systemic implications of increased intestinal permeability are further illustrated by observations in patients with advanced chronic liver disease, where hematological abnormalities—such as macrocytosis and vitamin B12/folate deficiency—have been directly associated with enhanced gut barrier dysfunction, dysbiosis, and malnutrition [61].

### 5.3. Endotoxemia

#### 5.3.1. Bacterial Translocation

Although increased intestinal permeability—commonly referred to as “leaky gut”—is a well-established feature of IBS, its relationship with endotoxemia remains paradoxical. Direct tissue studies have confirmed that colonic biopsies from IBS patients demonstrate significantly increased translocation of both live bacteria and LPS compared to healthy controls. Notably, this process is mediated by mast cell activation and proceeds via the transcellular route, as opposed to straightforward paracellular leakage [62,63]. However, this local translocation does not reliably translate into measurable systemic endotoxemia, as the majority of studies report no significant elevation in circulating LPS levels in IBS patients relative to healthy controls. This discrepancy is likely attributable to the efficient first-pass clearance of endotoxin by hepatic Kupffer cells. These findings suggest that, in IBS, bacterial product translocation represents a low-grade, intermittent phenomenon whose primary pathophysiological impact may be exerted locally—within the intestinal wall or the porto-hepatic circulation—rather than in the systemic compartment. Notably, gut-derived factors also play a crucial role in the development and progression of chronic liver diseases [64,65].

#### 5.3.2. Elevated Serum LPS and LPS-Binding Protein in IBS Patients

Accumulating evidence demonstrates that patients with IBS, particularly the IBS-D phenotype [66], exhibit significantly elevated circulating levels of LPS-binding protein (LBP) [67], and, in some studies, direct LPS measurement. It is important to note that LBP—an acute-phase protein that binds LPS—is considered a more sensitive and stable surrogate marker of endotoxin exposure than direct LPS measurement, as it reflects cumulative or intermittent translocation. These biomarkers serve as clinical indicators of bacterial translocation, suggesting that gut-derived endotoxins infiltrate the systemic circulation via altered tight junctions. This state of low-grade endotoxemia frequently correlates with both symptom severity and elevated pro-inflammatory cytokine profiles, thereby reinforcing the hypothesis that increased intestinal permeability (“leaky gut”) and the consequent host immune response to microbial antigens constitute central mechanisms underlying the inflammatory processes in IBS [68,69]. Therefore, although IBS patients demonstrate increased local bacterial translocation across the colonic mucosa via mast cell-dependent transcellular routes, this does not reliably translate into measurable systemic endotoxemia due to efficient hepatic first-pass clearance by Kupffer cells—yet paradoxically, multiple studies report significantly elevated serum LPS and LBP levels in IBS-D, suggesting that endotoxin escape may be intermittent, low-grade, or detectable only with sensitive biomarkers rather than direct LPS quantification, Consequently, the inconsistency in direct LPS elevation across IBS studies likely reflects methodological variability and efficient hepatic clearance, while consistently elevated LBP supports the presence of low-grade, intermittent endotoxemia—detectable via integrated biomarkers rather than direct LPS quantification—particularly in IBS-D [64,65,66,67].

### 5.4. Hepatic TLR4 Activation

Based on experimental evidence from animal models and cell culture systems, it is hypothesized that, the context of IBS-D, increased intestinal permeability facilitates the translocation of bacterial LPS from the gut lumen into the portal circulation, thereby enabling its delivery to the liver. Once in the liver, LPS activates TLR4 expressed on both Kupffer cells and hepatocytes, subsequently triggering downstream signaling cascades, including the nuclear factor kappa-B (NF-κB) pathway [23,70]. This transcriptional activation leads to an increased production of pro-inflammatory cytokines such as IL-6 and TNF-α [71]. In the liver, this chronic, low-grade TLR4-mediated inflammation drives key pathological features of hepatosteatosis: TNF-α and IL-6 promote insulin resistance in hepatocytes, increasing DNL and impairing fatty acid oxidation, while IL-6 further exacerbates steatosis by enhancing lipid droplet formation [72]. Concurrently, Kupffer cell activation perpetuates a cycle of hepatic inflammation and stellate cell pro-fibrotic priming [73]. Thus, in susceptible IBS patients with prolonged dysbiosis and gut barrier disruption, recurrent hepatic TLR4 activation by gut-derived LPS serves as a critical mechanistic link between the functional bowel disorder and the development or progression of MASLD [74].

It is important to emphasize that while the TLR4 signaling pathway is well-characterized in experimental models of steatosis, direct evidence for this pathway operating in IBS-MASLD patients is largely inferential. Most supportive data derive from animal models, in vitro hepatocyte cultures, or patients with other diseases (e.g., alcoholic liver disease, sepsis). The specific contribution of IBS-induced endotoxemia to hepatic TLR4 activation in humans remains an open question requiring dedicated investigation.

### 5.5. DNL and Reduced Fatty Acid Oxidation

**Figure 2:**
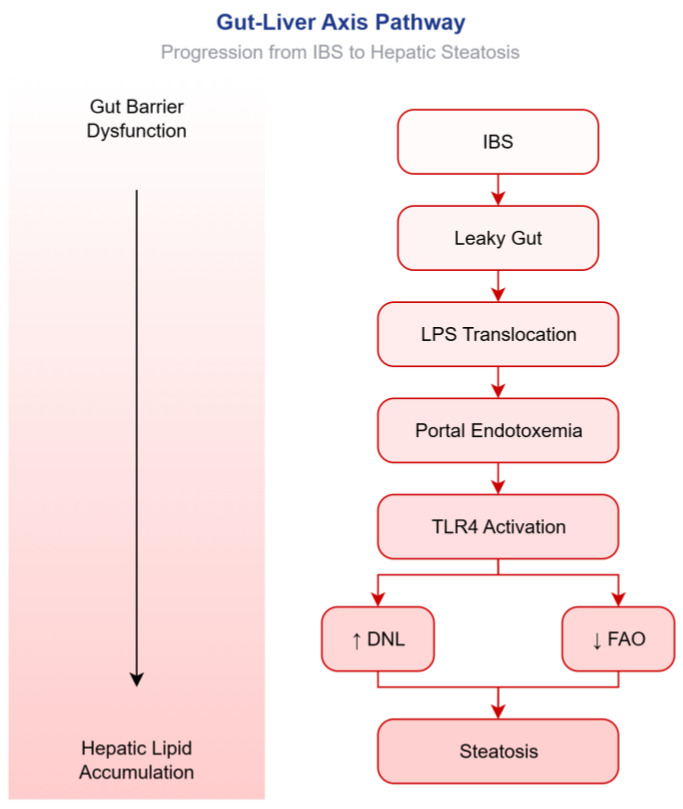
Proposed simplified schematic of the gut–liver axis in IBS-associated hepatic steatosis. IBS, irritable bowel syndrome; LPS, lipopolysaccharide; TLR4, toll-like receptor 4; DNL, de novo lipogenesis; FAO, fatty acid oxidation.

Human clinical studies have demonstrated that, in patients with irritable bowel syndrome (IBS), particularly those with the diarrhea-predominant subtype (IBS-D), disruptions in bile acid homeostasis and gut-derived signaling molecules contribute to hepatic metabolic dysregulation, thereby promoting triglyceride accumulation within the liver. A key mediator in this process is fibroblast growth factor 19 (FGF19), an ileal enterokine that normally suppresses hepatic DNL, while enhancing fatty acid oxidation and insulin sensitivity [75]. In subsets of patients with diarrhea-predominant IBS (IBS-D), deficient production or release of FGF19 leads to reduced signaling through the fibroblast growth factor receptor 4 (FGFR4)/klotho beta (KLB) pathway. This effectively derepresses hepatic DNL and impairs the lipid clearance mechanisms that normally prevent steatosis. Chronically, this state of metabolic dysregulation is exacerbated by pro-inflammatory cytokines, including TNF-α and IL-6, which further promote insulin resistance, upregulate DNL, and downregulate fatty acid β-oxidation [75,76]. The effect of increased DNL coupled with reduced fatty acid oxidation is the accumulation of free fatty acids and their subsequent esterification into triglycerides, which are then deposited as lipid droplets within hepatocytes. Thus, in susceptible IBS patients presenting with concurrent FGF19 deficiency and low-grade systemic inflammation, the imbalance between DNL and fatty acid oxidation establishes a permissive metabolic environment that favors the development or progression of hepatic steatosis [77,78]. The proposed stepwise cascade from IBS-driven gut barrier dysfunction to hepatic lipid accumulation is schematically outlined in Figure 2.

### 5.6. Secondary Mechanisms

#### 5.6.1. From Bile Acid Malabsorption to Hepatic Steatosis: The Role of Altered FXR Signaling in IBS-D

Human studies have established that, in patients with IBS-D who have underlying bile acid malabsorption (BAM), diminished reabsorption of bile acids in the terminal ileum results in altered signaling through the FXR [79,80]. Specifically, decreased ileal FXR activation results in lower FGF19 secretion, which in turn disinhibits hepatic CYP7A1 expression and promotes de novo bile acid synthesis [81]. Concomitantly, compromised FXR signaling within the liver, exacerbated by an altered bile acid pool and reduced FGF19 feedback, impairs hepatic lipid homeostasis by promoting DNL (via SREBP-1c dysregulation) and reducing VLDL export and fatty acid oxidation [81,82]. Consequently, this FXR-driven metabolic maladaptation can facilitate hepatic triglyceride accumulation, potentially contributing to MASLD in a subset of IBS-D patients with severe or long-standing BAM [81,83].

#### 5.6.2. Altered Firmicutes/Bacteroidetes Ratio and Gut-Derived Ethanol: Implications for Hepatic Injury

Emerging evidence identifies gut dysbiosis—specifically an altered Firmicutes-to-Bacteroidetes ratio—as a critical driver of endogenous ethanol production, thereby establishing a state of functional “auto-brewery” that directly contributes to hepatotoxicity [84]. While Firmicutes and Bacteroidetes constitute the dominant phyla in the healthy human gut, a compositional shift favoring certain fermentative members can profoundly impact the metabolic output of the microbiome. This dysbiotic environment promotes the proliferation of ethanol-producing bacteria, which ferment dietary carbohydrates into significant quantities of ethanol [85,86]. When this endogenous ethanol production exceeds the liver’s metabolic capacity, it reaches the portal circulation and induces hepatotoxicity through mechanisms strikingly similar to those of exogenous alcohol consumption, including oxidative stress, steatosis, and inflammation. This pathway from a “leaky gut” and microbial imbalance to liver pathology underscores the direct causal link between an altered Firmicutes/Bacteroidetes ratio, elevated endogenous ethanol, and the progression of liver disease [86].

These mechanisms represent biologically plausible pathways derived from in vitro studies, animal models, and extrapolation from other disease states. While these mechanisms provide a coherent framework for understanding how IBS might influence hepatic metabolism, it is important to recognize that direct evidence in humans with IBS-MASLD comorbidity is limited. Thus, these pathways should be interpreted as hypotheses requiring validation through prospective interventional studies, rather than as established causal mechanisms.

## 6. Bidirectionality–Does Steatosis Worsen IBS?

**Figure 3:**
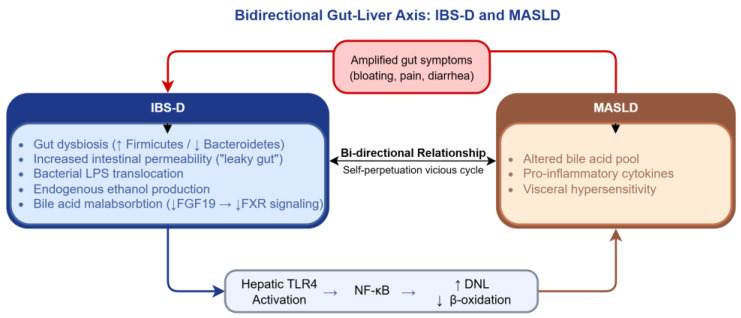
Proposed schematic of the bidirectional gut–liver axis in IBS-D and MASLD comorbidity. IBS-D, irritable bowel syndrome with diarrhea; MASLD, metabolic dysfunction-associated steatotic liver disease; LPS, lipopolysaccharide; FGF19, fibroblast growth factor 19; FXR, farnesoid X receptor; TLR4, toll-like receptor 4; NF-κB, nuclear factor kappa-light-chain-enhancer of activated B cells; DNL, de novo lipogenesis.

Hepatic steatosis may exacerbate IBS through several interconnected mechanisms, including altered bile acid metabolism, heightened visceral sensitivity, and the release of pro-inflammatory cytokines that influence the enteric nervous system. This hypothesis, while biologically plausible, remains speculative in the absence of prospective studies demonstrating that steatosis independently worsens IBS symptoms. Therefore, while steatosis does not necessarily worsen IBS symptoms in all patients, it likely amplifies symptom severity and complicates clinical outcomes in those with overlapping pathophysiological pathways. This bidirectional relationship may establish a self-perpetuating, vicious cycle: IBS promotes hepatic steatosis via mechanisms including gut dysbiosis, increased intestinal permeability (“leaky gut”), and altered bile acid metabolism; in turn, steatosis exacerbates IBS through the release of pro-inflammatory mediators and the induction of visceral hypersensitivity. Such forward-feeding loops—IBS leading to steatosis, which subsequently worsens IBS—suggest that hepatic steatosis not only coexists with but actively amplifies gastrointestinal symptoms. This pathophysiological interplay may explain the refractory clinical course observed in a subset of affected patients. Figure 3 provides a schematic representation of the self-perpetuating vicious cycle between IBS-D and MASLD.

## 7. Toward Clinical Application: Implications for Practice

### 7.1. Should IBS Patients Be Screened for MASLD?

**Figure 4:**
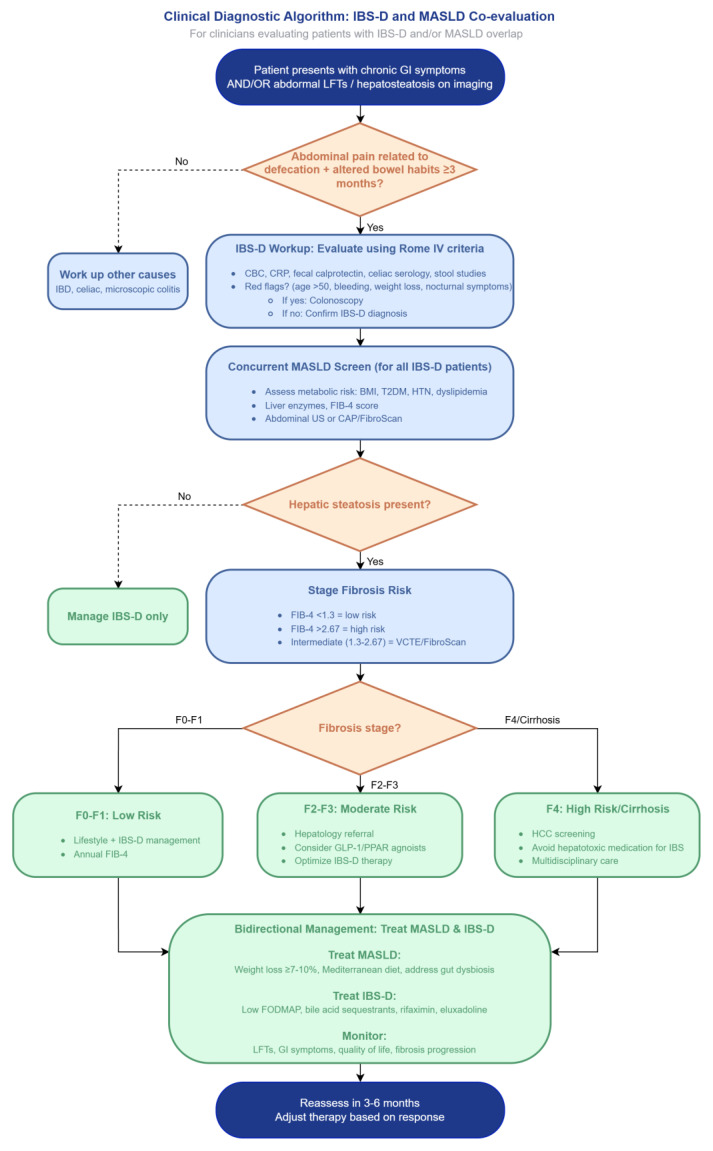
Clinical diagnostic algorithm for the co-evaluation and management of patients with overlapping IBS-D and MASLD. BMI, body mass index; CAP, controlled attenuation parameter; CBC, complete blood count; CRP, C-reactive protein; FIB-4, fibrosis-4 index; FODMAP, fermentable oligosaccharides, disaccharides, monosaccharides, and polyols; GI, gastrointestinal; GLP-1, glucagon-like peptide-1; HCC, hepatocellular carcinoma; HTN, hypertension; IBD, inflammatory bowel disease; IBS-D, irritable bowel syndrome with diarrhea; LFTs, liver function tests; MASLD, metabolic dysfunction-associated steatotic liver disease; PPAR, peroxisome proliferator-activated receptor; T2DM, type 2 diabetes mellitus; US, ultrasound; VCTE, vibration-controlled transient elastography.

Given the proposed bidirectional relationship and the potential self-perpetuating vicious cycle (IBS increases steatosis, and steatosis exacerbates IBS), selective screening for MASLD may be warranted in patients with IBS, particularly among those presenting with concomitant metabolic risk factors, including obesity, insulin resistance, or dyslipidemia. Although routine population wide screening is not currently recommended, IBS patients with refractory symptoms or elevated liver enzymes could benefit from hepatic assessment, for instance, via ultrasonography or transient elastography (FibroScan) [4]. Early detection of steatosis in this subgroup might allow for targeted interventions that break the vicious cycle, potentially improving both hepatic and gastrointestinal outcomes (Figure 4) [4,12].

### 7.2. Can Treating IBS Reverse Steatosis?

Effective management of IBS—specifically when targeting gut dysbiosis, visceral hypersensitivity, and intestinal permeability—may confer indirect benefits on hepatic steatosis by reducing systemic inflammation and modulating the gut–liver axis. Interventions such as dietary modifications (e.g., the low FODMAP diet), probiotics, or rifaximin have the potential to decrease bacterial translocation and endotoxemia, both of which are implicated in the pathogenesis of MASLD [87].

The integrated management of IBS and MASLD capitalizes on the shared pathophysiology of the gut–liver axis [88]. First-line therapy for both IBS and MASLD centers on lifestyle modification, with particular emphasis on adherence to a Mediterranean diet and gradual weight loss of 5–10% of total body weight. This approach not only reduces hepatic steatosis but also ameliorates IBS symptoms, including bloating and bowel habit irregularities. For patients with refractory IBS, a short-term low-FODMAP diet implemented under dietitian guidance is an effective strategy; however, it must be carefully applied to avoid compromising prebiotic fiber intake, which is beneficial for maintaining liver health [2]. Pharmacologically, treatment is symptom-driven for IBS (e.g., linaclotide for constipation, rifaximin for diarrhea), while emerging metabolic agents like glucagon-like peptide-1 (GLP-1) receptor agonists (semaglutide, tirzepatide) and the liver-directed therapy resmetirom offer potent options for progressive MASLD [2]. Notably, the use of select multi-strain probiotics or synbiotics has emerged as a promising adjunctive therapy capable of simultaneously improving intestinal permeability, reducing endotoxemia, and lowering liver enzyme levels. As such, this approach represents the most direct current example of targeted gut–liver axis modulation in clinical practice [89,90,91].

## 8. Limitations and Gaps of Current Evidence

A fundamental limitation of the current evidence base is the predominance of cross-sectional and observational cohort designs. While the mechanistic pathways described are biologically plausible, the available human data are insufficient to establish a causal relationship between IBS and MASLD as the preponderance of available evidence derives from single-timepoint observational designs, which inherently preclude the establishment of temporal precedence. Consequently, the directionality of the association between IBS and hepatic steatosis, and, by extension, a causal inference remains unsubstantiated.

The persistent threat of residual confounding cannot be overlooked, as diet and physical activity—key determinants of both hepatic steatosis and gastrointestinal symptomatology—are notoriously difficult to capture with sufficient granularity and to adjust for fully in observational analyses. To date, no randomized controlled trial has investigated whether therapeutic intervention for irritable bowel syndrome confers a preventive effect on the development or progression of hepatic steatosis, underscoring a critical gap in the interventional evidence base.

The lack of biopsy-proven MASLD across most IBS-related studies undermines diagnostic precision, as non-invasive modalities may misclassify hepatic fat content and cannot establish the definitive presence or grade of steatosis.

## 9. Toward a Causal Framework: Priorities for Future Investigation

Given the limitations of the current evidence base, future research must prioritize rigorous methodologies to move beyond association toward causal inference, such as: the adoption of prospective cohort designs to establish temporal precedence; the incorporation of biopsy-proven MASLD to ensure diagnostic precision; rigorous adjustment for dietary and physical activity confounders using validated instruments; and, most critically, the execution of randomized controlled trials to determine whether targeted IBS therapy can meaningfully modify hepatic steatosis risk. Beyond merely confirming the existence of an association between IBS and hepatic steatosis, future investigations should pivot toward mechanistic elucidation through microbiome-mediated mediation analysis. Specifically, longitudinal studies incorporating serial fecal sampling and shotgun metagenomic sequencing are urgently needed to identify which distinct bacterial taxa—or their functional metabolic products—statistically mediate the putative pathway from IBS to subsequent hepatic fat accumulation. Formal mediation frameworks—such as structural equation modeling or counterfactual-based approaches—could quantify the proportion of the total IBS effect on steatosis that operates through specific microbial signatures, while distinctly separating direct from indirect pathways. Identifying candidate mediators, including species that alter bile acid metabolism, short-chain fatty acid production, or intestinal barrier integrity, would not only establish a causal chain but also nominate novel microbiota-directed therapeutic targets. In the absence of such analyses, the gut–liver axis in IBS remains a proverbial black box: the association may be observed, yet its mechanistic architecture will remain fundamentally speculative.

## 10. Conclusions

There is moderate strength of evidence that IBS, particularly IBS-D, is an independent risk factor for hepatic steatosis, underscoring a clinically relevant gut–liver interaction. The proposed mechanism is biologically plausible via the leaky gut–endotoxemia–TLR4–lipogenesis pathway, but direct human evidence confirming this sequence of events in IBS-MASLD patients is limited. Clinical screening for MASLD in long-standing IBS-D patients is reasonable but not yet guideline-endorsed. The relationship is bidirectional, creating a potential vicious cycle. Consequently, future prospective and interventional studies are needed to establish causality and guide therapy. An integrated approach moves management from liver-centric to whole-patient care, with potential to slow MASLD progression and improve quality of life.

## Figures and Tables

**Table 1:** Evidence of MASLD prevalence in IBS patients.

Authors	Study Design	No. of Participants	Key Finding on MASLD Prevalence in IBS vs. Non-IBS
Sarmini et al. (2019) [10]	Observational study	84.4 million	Adjusted Odds Ratio: Patients with IBS were 3.2 times more likely to have MASLD (95% CI: 3.13–3.28, *p* < 0.001) after adjusting for factors like age, obesity, and diabetes.
Wu et al. (2022) [1]	Prospective cohort	396,838	Risk of Developing IBS: MASLD patients had a 13% higher risk of developing IBS over ~12 years (HR = 1.13). Severity Link: The risk of IBS increased with higher fatty liver scores (21% higher risk in the top quartile).
Ke et al. (2013) [11]	Cross-sectional	945	Prevalence: IBS was present in 23.2% of MASLD patients, but only 12.5% of those without MASLD (*p* < 0.01). Severity Link: MASLD severity correlated with IBS prevalence (mild: 11.3% → severe: 58.3%).

**Table 2:** Evidence of association between IBS and metabolic syndrome components.

Study, Year	Population & Design	Key Findings
Guo et al. (2014) [20]	1096 middle-aged adults (Japan), cross-sectional	IBS significantly related to MetS (OR 2.01) and elevated triglycerides (OR 1.50). This was the first population-based study to report this link.
Lee et al. (2016) [22]	83 IBS cases vs. 260 controls (Korea), cross-sectional	IBS patients had significantly higher BMI, waist circumference, and lipid levels. Prevalence of MetS was much higher in the IBS group (32.5% vs. 12.7%). Associations remained significant after controlling for potential confounding factors.
Abboud et al. (2025) [19]	221 Lebanese adults, cross sectional	MetS positively associated with total IBS (VAS) and specifically with the diarrhea-predominant (IBS-D) subscale. Individual MetS components (elevated blood pressure, fasting blood sugar, and waist circumference) were also linked to IBS-D
Nozu & Okumura (2022) [23]	Comprehensive Review	Summarizes evidence for pathophysiological commonality between IBS and MetS, focusing on shared mechanisms like ‘leaky gut’, dysbiosis, and inflammation (CRF-TLR4-proinflammatory cytokine signaling).

## Data Availability

No new data were created.

## References

[1] Wu S., Yuan C., Yang Z., Liu S., Zhang Q., Zhang S., Zhu S. (2022). Non-Alcoholic Fatty Liver Is Associated with Increased Risk of Irritable Bowel Syndrome: A Prospective Cohort Study. BMC Med..

[2] Zhou Y., Yang Z., Liu S., Xie S., Zhang Q., Zhang S., Zhu S., Wu S. (2025). Long-Term Risk of Irritable Bowel Syndrome Associated with MASLD, MASLD Type and Different Cardiometabolic Risk Factors: A Large-Scale Prospective Cohort Study. BMC Gastroenterol..

[3] Scalera A., Di Minno M.N.D., Tarantino G. (2013). What Does Irritable Bowel Syndrome Share with Non-Alcoholic Fatty Liver Disease?. World J. Gastroenterol..

[4] Ng J.J.J., Loo W.M., Siah K.T.H. (2023). Associations between Irritable Bowel Syndrome and Non-Alcoholic Fatty Liver Disease: A Systematic Review. World J. Hepatol..

[5] Passacatini L.C., Ilari S., Nucera S., Scarano F., Macrì R., Caminiti R., Serra M., Oppedisano F., Maiuolo J., Palma E. (2024). Multiple Aspects of Irritable Bowel Syndrome and the Role of the Immune System: An Overview of Systematic Reviews with a Focus on Polyphenols. Int. J. Mol. Sci..

[6] Purssell H., Bennett L., Street O., Hanley K.P., Hanley N., Vasant D.H., Athwal V.S. (2023). The Prevalence and Burden of Rome IV Bowel Disorders of Gut Brain Interaction in Patients with Non-Alcoholic Fatty Liver Disease: A Cross-Sectional Study. Sci. Rep..

[7] Camilleri M. (2019). Leaky Gut: Mechanisms, Measurement and Clinical Implications in Humans. Gut.

[8] Nakadate K., Saitoh H., Sakaguchi M., Miruno F., Muramatsu N., Ito N., Tadokoro K., Kawakami K. (2025). Advances in Understanding Lipopolysaccharide-Mediated Hepatitis: Mechanisms and Pathological Features. Curr. Issues Mol. Biol..

[9] Kapil S., Duseja A., Sharma B.K., Singla B., Chakraborti A., Das A., Ray P., Dhiman R.K., Chawla Y. (2016). Genetic Polymorphism in CD14 Gene, a Co-Receptor of TLR4 Associated with Non-Alcoholic Fatty Liver Disease. World J. Gastroenterol..

[10] Sarmini M., Asfari M., Alomari M., Al-khadra Y., Khoudari G., Dasarathy S., Mccullough A. (2019). Assessing the Relation Between Irritable Bowel Syndrome and Nonalcoholic Liver Disease: 478. Am. J. Gastroenterol..

[11] Ke Y., Yang T., Yao P. (2013). Association between Nonalcoholic Fatty Liver Disease and Irritable Bowel Syndrome in Populations Undergoing Health Examination in Urumqi. World J. Gastroenterol. (Chin. Ed.).

[12] Purssell H., Whorwell P.J., Athwal V.S., Vasant D.H. (2021). Non-Alcoholic Fatty Liver Disease in Irritable Bowel Syndrome: More than a Coincidence?. World J. Hepatol..

[13] Allam A.S., El Saeed K.M., Raafat K.M., Abozeid H.M. (2022). High Risk NAFLD among Patients with Irritable Bowel Syndrome: Frequency and Effect on Disease Severity. Egypt. J. Hosp. Med..

[14] Salim M.L.M., Youssef A.M.F., Elshafey E. (2023). Prevalence of Non-Alcoholic Fatty Liver Disease and Its Association with Severity in Patients with Irritable Bowel Syndrome. Egypt. J. Hosp. Med..

[15] Grover M., Kanazawa M., Palsson O.S., Chitkara D.K., Gangarosa L.M., Drossman D.A., Whitehead W.E. (2008). Small Intestinal Bacterial Overgrowth in Irritable Bowel Syndrome: Association with Colon Motility, Bowel Symptoms, and Psychological Distress. Neurogastroenterol. Motil..

[16] Šuran J., Pavlović N., Božić J., Kumrić M., Vukojević K., Filipović N., Radić B. (2026). IBS and SIBO: Gut Microbiota, Pathophysiology, and Non-Pharmacological Interventions. Antibiotics.

[17] Posserud I., Stotzer P.-O., Björnsson E.S., Abrahamsson H., Simrén M. (2007). Small Intestinal Bacterial Overgrowth in Patients with Irritable Bowel Syndrome. Gut.

[18] Efremova I., Maslennikov R., Poluektova E., Vasilieva E., Zharikov Y., Suslov A., Letyagina Y., Kozlov E., Levshina A., Ivashkin V. (2023). Epidemiology of Small Intestinal Bacterial Overgrowth. World J. Gastroenterol..

[19] Abboud M., Haidar S., Mahboub N., Mamo T., Papandreou D., Rizk R. (2025). Association between the Metabolic Syndrome and the Irritable Bowel Syndrome: A Cross-Sectional Study among a Sample of Lebanese Adults. Br. J. Nutr..

[20] Guo Y., Niu K., Momma H., Kobayashi Y., Chujo M., Otomo A., Fukudo S., Nagatomi R. (2014). Irritable Bowel Syndrome Is Positively Related to Metabolic Syndrome: A Population-Based Cross-Sectional Study. PLoS ONE.

[21] Schmelter F., Nowak L., Beier P., Tetzlaff-Lelleck V.V., Kordowski A., Föh B., Witt O., Schröder T., Günther U.L., Laumonnier Y. (2026). Cardiometabolic and Metabolic Profiles in Irritable Bowel Syndrome Associated with Type 2 Diabetes. Am. J. Physiol. Endocrinol. Metab..

[22] Lee S.-H., Kim K.-N., Kim K.-M., Joo N.-S. (2016). Irritable Bowel Syndrome May Be Associated with Elevated Alanine Aminotransferase and Metabolic Syndrome. Yonsei Med. J..

[23] Nozu T., Okumura T. (2022). Pathophysiological Commonality Between Irritable Bowel Syndrome and Metabolic Syndrome: Role of Corticotropin-Releasing Factor–Toll-like Receptor 4–Proinflammatory Cytokine Signaling. J. Neurogastroenterol. Motil..

[24] Liu J., Li C., Yang Y., Li J., Sun X., Zhang Y., Liu R., Chen F., Li X. (2025). Special Correlation between Diet and MASLD: Positive or Negative?. Cell Biosci..

[25] Palumbo P., Betto G.D., Menozzi R., Koek G.H., Buzzetti E. (2026). Pros and Cons of Different Dietary Patterns for the Treatment of Metabolic Dysfunction-Associated Steatotic Liver Disease. Metab.-Clin. Exp..

[26] Lomer M.C.E. (2024). The Low FODMAP Diet in Clinical Practice: Where Are We and What Are the Long-Term Considerations?. Proc. Nutr. Soc..

[27] Chu P., He Y., Hu F., Wang X. (2025). The Effects of Low FODMAP Diet on Gut Microbiota Regulation: A Systematic Review and Meta-Analysis. J. Food Sci..

[28] Lenover Moyer M.B., Shenk M.K. (2025). Physical Activity and Irritable Bowel Syndrome: The Role of Evolutionary Mismatch in Chronic Disease Risk. Am. J. Biol. Anthropol..

[29] Zhou Z., Li L.L., Wang C., Li S., Chen P., Huang J., Peng M. (2025). The Association between Sedentary Behavior and MASLD in Overweight and Obese Adults: Investigating the Role of Inflammatory Markers Using NHANES Data (2017–March 2020). Front. Nutr..

[30] Staudacher H.M., Black C.J., Teasdale S.B., Mikocka-Walus A., Keefer L. (2023). Irritable Bowel Syndrome and Mental Health Comorbidity—Approach to Multidisciplinary Management. Nat. Rev. Gastroenterol. Hepatol..

[31] Yang D., Zhao N., Zhang W., Peng S., Qin X., He W. (2025). Association between Mental Health and MASLD Molecular Insights through Metabolomics. Commun. Med..

[32] Gonzalez-Peña C., Uribe M., Chavez-Tapia N.C. (2026). Clinical and Molecular Implications of Antipsychotics in MASLD. Ann. Hepatol..

[33] Natalizio M., Nigam S., Rai V. (2025). Psychotropic Medications and Metabolic Side Effects. Explor. Endocr. Metab. Dis..

[34] Stryelkina M., Nguyen M., Vincent J.L. (2025). The Interplay between Sleep Disorders and MASLD: A Mini Review. Sleepmedicine.

[35] Olguner Eker Ö., Özsoy S., Eker B., Doğan H. (2017). Metabolic Effects of Antidepressant Treatment. Noro Psikiyatr. Ars..

[36] Imhann F., Bonder M.J., Vich Vila A., Fu J., Mujagic Z., Vork L., Tigchelaar E.F., Jankipersadsing S.A., Cenit M.C., Harmsen H.J.M. (2016). Proton Pump Inhibitors Affect the Gut Microbiome. Gut.

[37] Dean Y.E., Loayza Pintado J.J., Rouzan S.S., Nale L.L., Abbas A., Aboushaira A., Alkasajy F., Ghanem A.A., Patil V.M., Gordeyeva Y. (2025). The Relationship Between Irritable Bowel Syndrome and Metabolic Syndrome: A Systematic Review and Meta-Analysis of 49,662 Individuals. Endocrinol. Diabetes Metab..

[38] Raskov H., Burcharth J., Pommergaard H.-C., Rosenberg J. (2016). Irritable Bowel Syndrome, the Microbiota and the Gut-Brain Axis. Gut Microbes.

[39] Zhao Y., Zhu S., Dong Y., Xie T., Chai Z., Gao X., Dai Y., Wang X. (2024). The Role of Gut Microbiome in Irritable Bowel Syndrome: Implications for Clinical Therapeutics. Biomolecules.

[40] Min Y.W., Rezaie A., Pimentel M. (2022). Bile Acid and Gut Microbiota in Irritable Bowel Syndrome. J. Neurogastroenterol. Motil..

[41] Du L., Zhang Z., Zhai L., Xu S., Yang W., Huang C., Lin C., Zhong L.L.D., Bian Z., Zhao L. (2023). Altered Gut Microbiota-Host Bile Acid Metabolism in IBS-D Patients with Liver Depression and Spleen Deficiency Pattern. Chin. Med..

[42] Crocetta A., Giannelou M.-A., Benfante A., Castelli L., Koumbi L. (2025). Linking Psychological Stress to Epigenetic Regulation via the Gut–Liver–Brain Axis in Irritable Bowel Syndrome and Metabolic Dysfunction-Associated Fatty Liver Disease. Livers.

[43] Lee K.N., Lee O.Y. (2016). The Role of Mast Cells in Irritable Bowel Syndrome. Gastroenterol. Res. Pract..

[44] Camilleri M., Madsen K., Spiller R., Greenwood-Van Meerveld B., Verne G.N. (2012). Intestinal Barrier Function in Health and Gastrointestinal Disease. Neurogastroenterol. Motil..

[45] Vanuytsel T., Tack J., Farre R. (2021). The Role of Intestinal Permeability in Gastrointestinal Disorders and Current Methods of Evaluation. Front. Nutr..

[46] Wallon C., Yang P.-C., Keita A.V., Ericson A.-C., McKay D.M., Sherman P.M., Perdue M.H., Söderholm J.D. (2008). Corticotropin-Releasing Hormone (CRH) Regulates Macromolecular Permeability via Mast Cells in Normal Human Colonic Biopsies in Vitro. Gut.

[47] Livzan M.A., Gaus O.V. (2021). Fecal Zonulin as a Biomarker of Increased Intestinal Permeability in Patients with Irritable Bowel Syndrome (Narrative Review and Pilot Study Results). Dok. gastroenterol..

[48] Sturgeon C., Fasano A. (2016). Zonulin, a Regulator of Epithelial and Endothelial Barrier Functions, and Its Involvement in Chronic Inflammatory Diseases. Tissue Barriers.

[49] Bayoumi Hilal E.M., El-Ghandour A.M., Al- Ashram M.B., Zaki Mohamed A.M. (2024). Assessment of Serum Zonulin in Patients with Irritable Bowel Syndrome and Its Correlation with Stool Frequency. QJM.

[50] Linsalata M., Ignazzi A., D’Attoma B., Riezzo G., Mallardi D., Orlando A., Prospero L., Notarnicola M., De Nunzio V., Pinto G. (2024). Relationship between Markers of Gut Barrier Function and Erythrocyte Membrane PUFAs in Diarrhea-Predominant IBS Patients Undergoing a Low-FODMAP Diet. Nutrients.

[51] Gaus O.V., Livzan M.A. (2023). Zonulin Levels Are Associated with Cortisol, Dopamine, and Serotonin Levels in Irritable Bowel Syndrome. Exp. Clin. Gastroenterol..

[52] Philpott H., Nandurkar S., Lubel J., Gibson P.R. (2013). Alternative Investigations for Irritable Bowel Syndrome. J. Gastroenterol. Hepatol..

[53] Rao A.S., Camilleri M., Eckert D.J., Busciglio I., Burton D.D., Ryks M., Wong B.S., Lamsam J., Singh R., Zinsmeister A.R. (2011). Urine Sugars for in Vivo Gut Permeability: Validation and Comparisons in Irritable Bowel Syndrome-Diarrhea and Controls. Am. J. Physiol.-Gastrointest. Liver Physiol..

[54] McOmber M., Rafati D., Cain K., Devaraj S., Weidler E.M., Heitkemper M., Shulman R.J. (2020). Increased Gut Permeability in First-Degree Relatives of Children with Irritable Bowel Syndrome or Functional Abdominal Pain. Clin. Gastroenterol. Hepatol..

[55] Ghosh S.S., Wang J., Yannie P.J., Ghosh S. (2020). Intestinal Barrier Dysfunction, LPS Translocation, and Disease Development. J. Endocr. Soc..

[56] Del Valle-Pinero A.Y., Van Deventer H.E., Fourie N.H., Martino A.C., Patel N.S., Remaley A.T., Henderson W.A. (2013). Gastrointestinal Permeability in Patients with Irritable Bowel Syndrome Assessed Using a Four Probe Permeability Solution. Clin. Chim. Acta.

[57] Awad K., Barmeyer C., Bojarski C., Nagel O., Lee I.-F.M., Schweiger M.R., Schulzke J.-D., Bücker R. (2023). Epithelial Barrier Dysfunction in Diarrhea-Predominant Irritable Bowel Syndrome (IBS-D) via Downregulation of Claudin-1. Cells.

[58] Ishimoto H., Oshima T., Sei H., Yamasaki T., Kondo T., Tozawa K., Tomita T., Ohda Y., Fukui H., Watari J. (2017). Claudin-2 Expression Is Upregulated in the Ileum of Diarrhea Predominant Irritable Bowel Syndrome Patients. J. Clin. Biochem. Nutr..

[59] Garcia-Hernandez V., Quiros M., Nusrat A. (2017). Intestinal Epithelial Claudins: Expression and Regulation in Homeostasis and Inflammation. Ann. N. Y. Acad. Sci..

[60] Linsalata M., Riezzo G., Clemente C., D’Attoma B., Russo F. (2020). Noninvasive Biomarkers of Gut Barrier Function in Patients Suffering from Diarrhea Predominant-IBS: An Update. Dis. Markers.

[61] Marginean C.M., Pirscoveanu D., Popescu M., Docea A.O., Radu A., Popescu A.I.S., Vasile C.M., Mitrut R., Marginean I.C., Iacob G.A. (2023). Diagnostic Approach and Pathophysiological Mechanisms of Anemia in Chronic Liver Disease—An Overview. Gastroenterol. Insights.

[62] Undseth R., Berstad A., Valeur J. (2016). Systemic Symptoms in Irritable Bowel Syndrome: An Investigative Study on the Role of Enterocyte Disintegrity, Endotoxemia and Inflammation. Mol. Med. Rep..

[63] Wilcz-Villega E.M., McClean S., O’Sullivan M.A. (2013). Mast Cell Tryptase Reduces Junctional Adhesion Molecule-A (JAM-A) Expression in Intestinal Epithelial Cells: Implications for the Mechanisms of Barrier Dysfunction in Irritable Bowel Syndrome. Am. J. Gastroenterol..

[64] An L., Wirth U., Koch D., Schirren M., Drefs M., Koliogiannis D., Nieß H., Andrassy J., Guba M., Bazhin A.V. (2022). The Role of Gut-Derived Lipopolysaccharides and the Intestinal Barrier in Fatty Liver Diseases. J. Gastrointest. Surg..

[65] Twardowska A., Makaro A., Binienda A., Fichna J., Salaga M. (2022). Preventing Bacterial Translocation in Patients with Leaky Gut Syndrome: Nutrition and Pharmacological Treatment Options. Int. J. Mol. Sci..

[66] Zhou Q., Verne M.L., Fields J.Z., Lefante J.J., Basra S., Salameh H., Verne G.N. (2019). Randomised Placebo-Controlled Trial of Dietary Glutamine Supplements for Postinfectious Irritable Bowel Syndrome. Gut.

[67] Soufan F., Ghosson A., Jaber R., Ghandour A., Uwishema O. (2025). The Gut-Brain Axis in Irritable Bowel Syndrome: Implementing the Role of Microbiota and Neuroimmune Interaction in Personalized Prevention-A Narrative Review. Health Sci. Rep..

[68] Sheikhesmaili F., Jalili A., Taghizadeh E., Fakhari S., Jalili K., Ghaderi E., Rahimi E. (2021). The CCL28 Levels Are Elevated in the Serum of Patients with Irritable Bowel Syndrome and Associated with the Clinical Symptoms. Am. J. Clin. Exp. Immunol..

[69] Ullah A., Shen B. (2026). Modulating the Gut-Liver Axis: Anti-Inflammatory Mechanisms of Probiotics and Prebiotics in MASLD. Probiotics Antimicro. Prot.

[70] Hu L., Cheng Z., Chu H., Wang W., Jin Y., Yang L. (2024). TRIF-Dependent Signaling and Its Role in Liver Diseases. Front. Cell Dev. Biol..

[71] Ren G., Bai C., Yi S., Cong Q., Zhu Y. (2023). Mechanisms and Therapeutic Strategies for MAFLD Targeting TLR4 Signaling Pathways. J. Innate Immun..

[72] De Cól J.P., de Lima E.P., Pompeu F.M., Cressoni Araújo A., de Alvares Goulart R., Bechara M.D., Laurindo L.F., Méndez-Sánchez N., Barbalho S.M. (2024). Underlying Mechanisms behind the Brain-Gut-Liver Axis and Metabolic-Associated Fatty Liver Disease (MAFLD): An Update. Int. J. Mol. Sci..

[73] Szabo G., Bala S. (2010). Alcoholic Liver Disease and the Gut-Liver Axis. World J. Gastroenterol..

[74] Zhou M., Lv J., Chen X., Shi Y., Chao G., Zhang S. (2025). From Gut to Liver: Exploring the Crosstalk between Gut-Liver Axis and Oxidative Stress in Metabolic Dysfunction-Associated Steatotic Liver Disease. Ann. Hepatol..

[75] Weaver M.J., McHenry S.A., Sayuk G.S., Gyawali C.P., Davidson N.O. (2020). Bile Acid Diarrhea and NAFLD: Shared Pathways for Distinct Phenotypes. Hepatol. Commun..

[76] Chen Z., Yu R., Xiong Y., Du F., Zhu S. (2017). A Vicious Circle between Insulin Resistance and Inflammation in Nonalcoholic Fatty Liver Disease. Lipids Health Dis..

[77] Alvarez-Sola G., Uriarte I., Latasa M.U., Fernandez-Barrena M.G., Urtasun R., Elizalde M., Barcena-Varela M., Jiménez M., Chang H.C., Barbero R. (2017). Fibroblast Growth Factor 15/19 (FGF15/19) Protects from Diet-Induced Hepatic Steatosis: Development of an FGF19-Based Chimeric Molecule to Promote Fatty Liver Regeneration. Gut.

[78] Tian H., Zhang S., Liu Y., Wu Y., Zhang D. (2023). Fibroblast Growth Factors for Nonalcoholic Fatty Liver Disease: Opportunities and Challenges. Int. J. Mol. Sci..

[79] Jia W., Xie G., Jia W. (2018). Bile Acid-Microbiota Crosstalk in Gastrointestinal Inflammation and Carcinogenesis. Nat. Rev. Gastroenterol. Hepatol..

[80] Camilleri M. (2015). Bile Acid Diarrhea: Prevalence, Pathogenesis, and Therapy. Gut Liver.

[81] Walters J.R.F., Tasleem A.M., Omer O.S., Brydon W.G., Dew T., le Roux C.W. (2009). A New Mechanism for Bile Acid Diarrhea: Defective Feedback Inhibition of Bile Acid Biosynthesis. Clin. Gastroenterol. Hepatol..

[82] Arab J.P., Karpen S.J., Dawson P.A., Arrese M., Trauner M. (2017). Bile Acids and Nonalcoholic Fatty Liver Disease: Molecular Insights and Therapeutic Perspectives. Hepatology.

[83] Fuchs C., Claudel T., Trauner M. (2013). Bile Acid-Mediated Control of Liver Triglycerides. Semin. Liver Dis..

[84] Ballway J.W., Song B.-J. (2021). Translational Approaches with Antioxidant Phytochemicals against Alcohol-Mediated Oxidative Stress, Gut Dysbiosis, Intestinal Barrier Dysfunction, and Fatty Liver Disease. Antioxidants.

[85] Meijnikman A.S., Nieuwdorp M., Schnabl B. (2024). Endogenous Ethanol Production in Health and Disease. Nat. Rev. Gastroenterol. Hepatol..

[86] Gu Z., Wu Y., Wang Y., Sun H., You Y., Piao C., Liu J., Wang Y. (2020). Lactobacillus Rhamnosus Granules Dose-Dependently Balance Intestinal Microbiome Disorders and Ameliorate Chronic Alcohol-Induced Liver Injury. J. Med. Food.

[87] Marginean C.M., Pirscoveanu D., Cazacu S.M., Popescu M.S., Marginean I.C., Iacob G.A., Popescu M. (2024). Non-Alcoholic Fatty Liver Disease, Awareness of a Diagnostic Challenge—A Clinician’s Perspective. Gastroenterol. Insights.

[88] Abenavoli L., Spagnuolo R., Scarlata G.G.M., Gambardella M.L., Boccuto L., Méndez-Sánchez N., Luzza F. (2024). Metabolic Dysfunction-Associated Steatotic Liver Disease in Patients with Inflammatory Bowel Diseases: A Pilot Study. Life.

[89] Marginean I.C., Cazacu S.M., Popescu M., Iacob G.A., Sandulescu L.D., Iordache S., Marginean C.M., Vere C.C. (2025). The Vicious Circle of Metabolic Dysfunction-Associated Steatotic Liver Disease When Micronutrient Deficiency Drives Microbial Imbalance and Liver Injury. Life.

[90] Rotaru A., Stafie R., Stratina E., Zenovia S., Nastasa R., Minea H., Huiban L., Cuciureanu T., Muzica C., Chiriac S. (2025). Lean MASLD and IBD: Exploring the Intersection of Metabolic Dysfunction and the Gut-Liver Axis. Life.

[91] Marginean C.M., Popescu M., Drocas A.I., Cazacu S.M., Mitrut R., Marginean I.C., Iacob G.A., Popescu M.S., Docea A.O., Mitrut P. (2023). Gut–Brain Axis, Microbiota and Probiotics—Current Knowledge on Their Role in Irritable Bowel Syndrome: A Review. Gastrointest. Disord..

